# A Systematic Review and Meta-Analysis of Cannabis Use Frequency and Metabolic Dysfunction-Associated Steatotic Liver Disease: Scapegoat or Healer?

**DOI:** 10.7759/cureus.93183

**Published:** 2025-09-25

**Authors:** Néstor Israel Quinapanta Castro, Abdel Bermúdez-del Sol

**Affiliations:** 1 Department of Research, Regional Autonomous University of the Andes (UNIANDES), Ambato, ECU; 2 Department of Postgraduate Studies, Regional Autonomous University of the Andes (UNIANDES), Ambato, ECU; 3 Department of Pharmacology, Regional Autonomous University of the Andes (UNIANDES), Ambato, ECU

**Keywords:** cannabis abuse, cannabis use, chronic cannabis use, fatty liver disease, metabolic dysfunction-associated steatotic liver disease

## Abstract

Potential associations have been investigated between metabolic dysfunction-associated steatotic liver disease (MASLD), formerly known as non-alcoholic fatty liver disease, and cannabis use. This study aimed to determine the association between cannabis use frequency and MASLD. Up to January 2025, the evidence from PubMed, Scopus, and Web of Science was synthesized in this systematic review and meta-analysis, which was registered in PROSPERO (CRD42025025065) and followed the Preferred Reporting Items for Systematic reviews and Meta-Analyses (PRISMA) guidelines. Of the 711 initial records, 11 observational studies involving 5,968,702 individuals met the inclusion criteria. A pooled analysis revealed that cannabis use was associated with a reduced risk of hepatic steatosis (OR = 0.58; 95% CI: 0.42-0.81; p = 0.002; I² = 97%). The subgroup analysis revealed a protective association for past users (OR = 0.84; 95% CI: 0.77-0.93) and occasional users (OR = 0.35; 95% CI: 0.20-0.64), with no significant association observed for frequent users. The study revealed that cannabis users exhibited a decline in both the fatty liver index (mean difference (MD) = -11.02) and the BMI (MD = -1.89 kg/m²). However, the findings did not show any statistically significant changes in liver fat (%), transaminases (aspartate aminotransferase and alanine aminotransferase), and triglycerides. A risk-of-bias assessment identified notable methodological limitations. Overall, the findings suggest a strong association between cannabis use and MASLD, though causality cannot be established.

## Introduction and background

Metabolic dysfunction-associated steatotic liver disease (MASLD), formerly known as non-alcoholic fatty liver disease [1], is the most common chronic liver disease worldwide [2-4]. Its prevalence has increased due to the popularity of Western diets and sedentary lifestyles [5]. The condition ranges from simple steatosis to non-alcoholic steatohepatitis (NASH) [1-3]. As part of the pathophysiological progression of MASLD, NASH represents a stage characterized by hepatic fat accumulation, sustained inflammation, and hepatocellular injury [5,6]. This can eventually lead to cirrhosis and the need for a liver transplant [5,6].

The overall incidence of EHMA is 47 cases per 1,000 people, occurring more frequently in men [7]. There was a significant 50.4% increase in its prevalence, rising from 25.26% between 1990 and 2006 to 38.00% between 2016 and 2019 (p < 0.001) [8]. The highest prevalence was observed in Latin America at 44.37% (30.66%-59.00%) [8].

MASLD is increasingly affecting younger people, which increases the risk of serious complications [3]. Due to changes in diet, urbanization, and the growth of obesity and type 2 diabetes, particularly in developing countries, the number of cases is expected to rise worldwide [3]. It is a public health problem associated with obesity, metabolic syndrome, and insulin resistance [9,10]. It results in the accumulation of fat in the liver and inflammation [9,10]. It is the hepatic manifestation of a systemic disease associated with type 2 diabetes and cardiovascular and renal disease [10].

Nowadays, it is understood that cannabis has emerged as a potential modulator of hepatic metabolism. The hepatic endocannabinoid system plays a key role in the accumulation of fat in the liver through cannabinoid receptor type 1 (CB1R) and cannabinoid receptor type 2 (CB2R) receptors [9]. Activation of the cannabinoid system has been linked to conditions such as cirrhosis and steatosis and also influences appetite regulation and energy metabolism [11]. The hepatic cannabinoid system is activated in MASLD, contributing to steatosis and insulin resistance [12]. CB1R is involved in the development of obesity and hepatic steatosis by stimulating de novo fatty acid synthesis [11]. CB1R expression is increased in fatty liver, and CB2R is involved in inflammation and hepatic fibrogenesis [11].

However, its management remains challenging due to its complexity [13]. Resmetirom is the first drug to be approved for treating MASLD with fibrosis, while the efficacy of other drugs is still under evaluation [13]. Meanwhile, research [14,15] suggests that cannabis users are less likely to have diabetes, obesity, and hyperlipidemia, which are closely related to MASLD.

Preclinical research, both in vivo and in vitro, has provided evidence supporting the therapeutic potential of cannabidiol (CBD) in a wide range of liver disorders [1]. Nonetheless, the underlying mechanisms remain insufficiently clarified, and the current scarcity of clinical data constrains their translation into routine clinical practice [1]. Contradictory evidence on the impact of cannabis on liver disease has been reported [16]. Cannabinoid receptors CB1 and CB2 are sparsely expressed in a healthy liver but overexpressed in chronic liver disease, potentially influencing the progression of steatosis and fibrosis [16].

This study aimed to determine the association between cannabis use frequency and MASLD, based on evidence obtained from a systematic review and meta-analysis of the literature.

## Review

Methods

A systematic peer review and meta-analysis of the specialized academic literature was performed in the PubMed/MEDLINE, Scopus, and Web of Science databases. This meta-analysis followed the Preferred Reporting Items for Systematic reviews and Meta-Analyses (PRISMA) protocol.

Search Strategy

A systematic search was conducted using the Population, Intervention, Comparison, Outcome (PICO) strategy, focusing on the following main question: What is the association between cannabis use and metabolic dysfunction-associated steatotic liver disease?, based on evidence obtained from a systematic review and meta-analysis of the literature. In April 2024, the first searches were conducted using a combination of Medical Subject Headings (MeSH): “Cannabis”, “Cannabinoids”, “Marijuana Use”, “Nonalcoholic Fatty Liver Disease”, and “Fatty Liver”.

Selection Criteria 

Quantitative, prospective, and retrospective observational studies, as well as cross-sectional studies, were included. Additionally, studies published in English or Spanish between 2005 and January 2025 should be considered. People over 18 years of age who use or consume cannabinoids, in any form, dose, or frequency, either for medical or recreational reasons, were included in the study. Editorials, letters to the editor, case series, and studies with nonhuman samples and in vitro tissue were excluded. Studies that addressed alcoholic fatty liver disease were also ruled out.

Primary and Secondary Outcomes

The primary outcomes of this review focused on the potential effect of cannabis use on the risk of hepatic steatosis, with prevalence assessed through imaging techniques, biochemical markers, or liver biopsy. The analysis also considered the fatty liver index (FLI) and BMI as additional indicators of liver health. Secondary outcomes included measurements of hepatic fat percentage and serum levels of alanine aminotransferase (ALT), aspartate aminotransferase (AST), and triglycerides.

Data Extraction and Synthesis 

Data extraction was performed using a form based on the Cochrane Consumers and Communication Group template for quality assessment and evidence synthesis. The form was adapted to collect all relevant information on the included studies and their results. The following data were extracted: authors, years, database consulted, journal of publication, date of publication, location of the study, type of article, DOI, original title, full abstracts, methodology applied, and results obtained. Two reviewers (NQC and ABS) independently extracted data, with disagreements resolved through consensus or consultation with an expert.

Each outcome’s findings were described in a narrative in the data synthesis. The quality of evidence will be assessed using the Grading of Recommendations Assessment, Development and Evaluation (GRADE) method, considering characteristics such as risk of bias, inconsistency, indirectness, imprecision, and publication bias.

Analysis Methods

The exposure impact measures included ORs with 95% CIs for dichotomous data and mean difference (MD) and standardized MD (SMD) for continuous data, depending on the consistency of the outcome measures.

The random-effects model was used for analyses with high heterogeneity (I² >50%) or interstudy variations in design or population. Conversely, the fixed-effects model was used for subgroups with low heterogeneity, analyses with few studies, or when a large, high-quality study predominated in the statistical weighting. Heterogeneity was assessed using Tau², Chi², and I² statistics. All analyses were performed using Review Manager (RevMan) version 5.7.0 (The Cochrane Collaboration, Copenhagen, Denmark). The subgroup analysis included the frequency of use, as well as current and past use of cannabis, in the meta-analysis.

Risk of Bias Assessment

Risk of bias was independently assessed by two reviewers (NQC and ABS), with disagreements resolved through consensus or consultation with an expert. The Risk of Bias in Non-Randomized Exposure Studies (ROBINS-E) tool was employed to evaluate the risk of bias in observational studies. The overall risk of bias was classified as low, high, or uncertain.

Register

This systematic review is registered in PROSPERO under the registration number CRD42025025065. Additionally, the findings were presented as a scientific poster at the ESCI 2025 Annual Scientific Meeting of the European Society for Clinical Investigation, held on May 21-23, 2025.

Results

**Figure 1:**
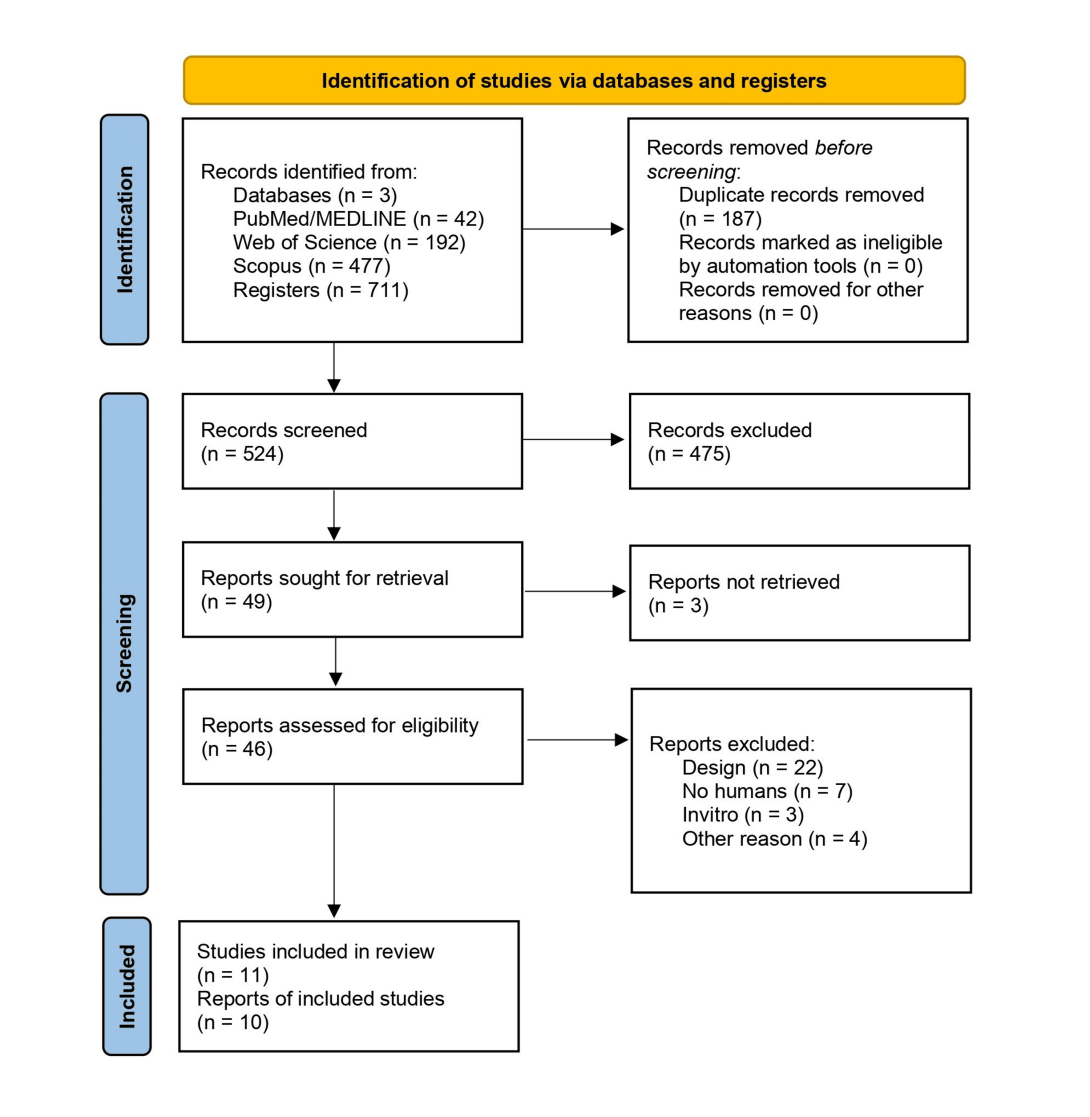
PRISMA flow diagram PRISMA, Preferred Reporting Items for Systematic reviews and Meta-Analyses

A total of 711 records were identified, 187 of which were eliminated due to duplication. Following an initial evaluation of the remaining 524 records, 475 were excluded because they did not meet the inclusion criteria. Of the 49 full texts reviewed, three could not be retrieved. Ultimately, 46 studies were evaluated, and 36 were excluded due to methodological or eligibility issues. Finally, 11 eligible studies were included, published across 10 scientific articles [17-26]. The process is detailed in the PRISMA diagram (Figure 1).

Table 1 presents the characteristics of the included studies. The mean (SD) age of the 5,968,702 participants was 41.29 (8.76) years. The studies were published between 2008 and 2023. Geographically, six of the 11 studies (55%) were conducted in North America [18,20,22,24,26], four (36%) in Europe [17,19,21,23], and one (9%) in Oceania [25]. In terms of study design, most were cross-sectional (n = 7), followed by retrospective (n = 2) and prospective (n = 2) studies.

**Table 1:** Baseline characteristics of the included studies ALT, alanine aminotransferase; FLI, fatty liver index; ICD-9: International Classification of Diseases, 9th Revision; MRS, magnetic resonance spectroscopy; NHANES, National Health and Nutrition Examination Survey; VCTE, vibration-controlled transient elastography

Author(s)	Country	Year	Study design	Diagnostic method	Baseline BMI (kg/m²)	Age (years)	Sex
Vázquez-Bourgon et al. [17]	Spain	2019	Prospective cohort	FLI	Nonusers: 33.7 (9.9); users: 25.2 (6.0)	30.4 (9.5)	217 males, 173 females
Liu et al. [18]	Canada	2014	Retrospective cohort	Liver biopsy	Not specified	Users: 43.9 ± 9.1; nonusers: 46.7 ± 8.1	265 males, 112 females
Hézode et al. [19]	France	2008	Cross-sectional	Liver biopsy	24.8 (4.1)	45.1 (10.9)	223 males, 92 females
Du et al. [20]	USA	2023	Cross-sectional, probabilistic	VCTE	Not specified	39.97 ± 11.65	1,287 males, 1,335 females
Barré et al. [21]	France	2021	Multicenter prospective cohort	FLI	Nonusers: 22.0; users: 20.9	47.8 ± 4.5	69.7% males, 30.3% females
Kim et al. [22] (NHANES)	USA	2017	Cross-sectional	ALT threshold	Never: 29.2 ± 0.1; former: 28.8 ± 0.1; current: 27.3 ± 0.3	39.6	7,040 males, 7,040 females
Kim et al. [22] (NHANES III)	USA	2017	Cross-sectional	Abdominal ultrasound	Never: 27.1 ± 0.2; former: 26.0 ± 0.2; current: 25.7 ± 0.4	37.6	4,002 males, 4,284 females
Nordmann et al. [23]	France	2017	Multicenter cross-sectional	Abdominal ultrasound	Not specified	44.9 (95% CI: 44.5-45.4)	585 males, 253 females
Adejumo et al. [24]	USA	2017	Retrospective case-control	Discharge diagnosis (ICD-9: 571.8)	Not specified	Not specified	2,373,361 males, 3,459,451 females
Stuart et al. [25]	New Zealand	2020	Cross-sectional	MRS	Never: 26.8; non-regular: 27.9; regular: 34.3	Never: 58; non-regular: 52; regular: 54	83 males, 37 females
Muniyappa et al. [26]	USA	2013	Cross-sectional, case-control	MRS	27 ± 6	27 ± 8	18 males, 12 females per group

Hepatic steatosis was assessed using imaging techniques, such as ultrasound, elastography, and magnetic resonance spectroscopy [20,22,23,25,26]; biochemical indices, including the FLI, liver biopsy, and serum ALT levels [18,19,21,22]; and diagnostic codes (ICD-9-MC 571.8) [24], as detailed in Table 1.

Primary Outcomes

**Figure 2:**
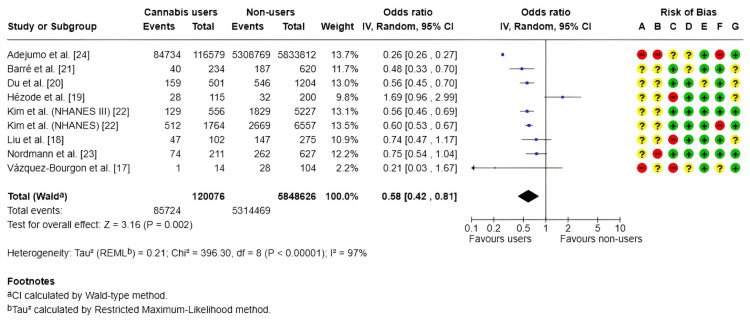
Forest plot and risk of bias: MASLD risk Random, random-effects model; + Low risk of bias; ? Some concerns; - High/very high risk of bias; ᵃ CI calculated using the Wald-type method; ᵇ Tau² calculated using the restricted maximum-likelihood method Risk of bias legend: (A) Risk of bias due to confounding; (B) Risk of bias arising from measurement of the exposure; (C) Risk of bias in selection of participants into the study (or into the analysis); (D) Risk of bias due to post-exposure interventions; (E) Risk of bias due to missing data; (F) Risk of bias arising from measurement of the outcome; (G) Risk of bias in the selection of the reported result IV, inverse variance; MASLD, metabolic dysfunction-associated steatotic liver disease References: [17-24]

MASLD: The nine studies [17-24] included in this meta-analysis indicated that individuals who used cannabis had a lower likelihood of presenting hepatic steatosis compared with nonusers. The pooled OR was 0.58 (95% CI: 0.42-0.81; p = 0.002), as shown in Figure 2, suggesting a potential protective association between cannabis use and this liver condition. Nonetheless, three individual studies crossed the null value, introducing some inconsistency in the interpretation of the results.

A high level of heterogeneity was observed among the studies (I² = 97%), reflecting substantial variability in the findings. This heterogeneity may be explained by methodological differences, variations in study populations, and clinical diversity. Furthermore, several studies exhibited high or unclear risks of bias, particularly in domains related to participant selection and outcome assessment.

Subgroup analysis: The included studies [17-24] were classified according to the type and frequency of cannabis use, encompassing current users, heavy users (≥4 days/week), moderate users, and former users. While some studies compared only current users with nonusers, others differentiated between levels of use (moderate or heavy) or included multiple exposure categories, also accounting for past use.

**Figure 3:**
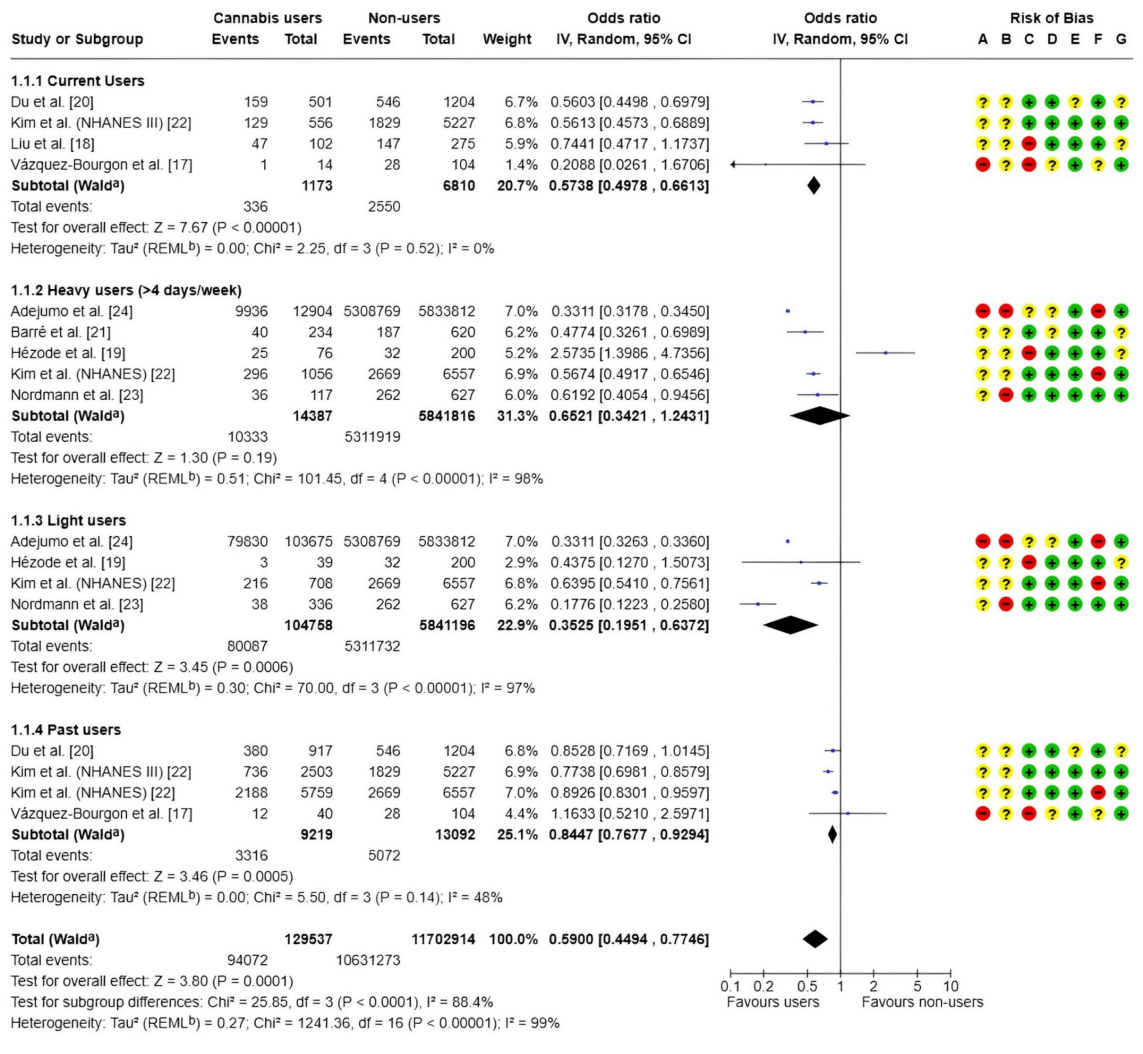
Forest plot and risk of bias: subgroup analysis of MASLD risk Random: random-effects model; + Low risk of bias; ? Some concerns; - High/very high risk of bias; ᵃ CI calculated using the Wald-type method; ᵇ Tau² calculated using the restricted maximum-likelihood method Risk of bias legend: (A) Risk of bias due to confounding; (B) Risk of bias arising from measurement of the exposure; (C) Risk of bias in selection of participants into the study (or into the analysis); (D) Risk of bias due to post-exposure interventions; (E) Risk of bias due to missing data; (F) Risk of bias arising from measurement of the outcome; (G) Risk of bias in the selection of the reported result IV, inverse variance; MASLD, metabolic dysfunction-associated steatotic liver disease References: [17-24]

The meta-analysis demonstrated that cannabis use was generally associated with a reduced likelihood of developing hepatic steatosis. The pooled effect estimate indicated a statistically significant reduction in risk among cannabis users compared with nonusers, with an OR of 0.59 (95% CI: 0.45-0.77; p < 0.00001), as presented in Figure 3.

Subgroup analyses based on frequency and timing of cannabis use revealed that current users experienced a significant reduction in the risk of hepatic steatosis (OR = 0.57; 95% CI: 0.5-0.66), with no observed heterogeneity between studies (I² = 0%). Light users also demonstrated a protective effect, with an OR of 0.35 (95% CI: 0.20-0.64); however, substantial heterogeneity was present (I² = 97%). Among heavy users (≥4 days per week), no statistically significant association was identified (OR = 0.65; 95% CI: 0.34-1.24), and high heterogeneity (I² = 98%) limited the interpretability of results for this subgroup. Ex-users exhibited a modestly reduced risk of steatosis (OR = 0.84; 95% CI: 0.77-0.93), although moderate heterogeneity was observed (I² = 48%).

Overall, a high degree of heterogeneity was present across all studies (I² = 99%), likely attributable to methodological, clinical, and population differences. The risk of bias assessment further highlighted important limitations, particularly in domains related to participant selection and exposure measurement.

**Figure 4:**
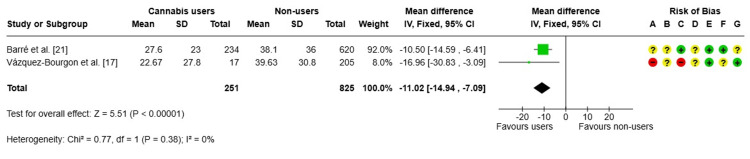
Forest plot and risk of bias: MDs in FLI Fixed: fixed-effects model; + Low risk of bias; ? Some concerns; - High/very high risk of bias Risk of bias legend: (A) Risk of bias due to confounding; (B) Risk of bias arising from measurement of the exposure; (C) Risk of bias in selection of participants into the study (or into the analysis); (D) Risk of bias due to post-exposure interventions; (E) Risk of bias due to missing data; (F) Risk of bias arising from measurement of the outcome; (G) Risk of bias in the selection of the reported result FLI, fatty liver index; IV, inverse variance; MD, mean difference References: [17,21]

FLI: The findings [17,21] demonstrated that individuals who currently used cannabis exhibited a substantial reduction in FLI values compared to nonusers (MD: -11.02 points), as illustrated in Figure 4. This association was statistically significant and consistent across studies (I² = 0%). However, these results should be interpreted with caution due to the potential for high or uncertain risk of bias across multiple methodological domains, particularly in one of the included studies.

**Figure 5:**
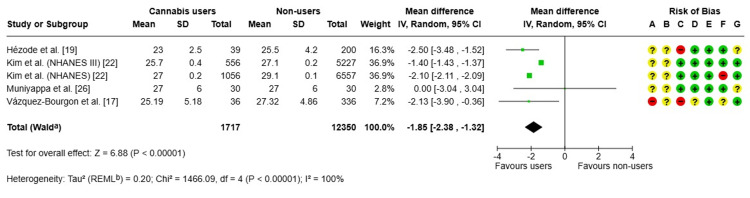
Forest plot and risk of bias: MDs in BMI (kg/m²) IV, inverse variance; Random, random-effects model; + Low risk of bias; ? Some concerns; - High/very high risk of bias; ᵃ CI calculated using the Wald-type method; ᵇ Tau² calculated using the restricted maximum-likelihood method Risk of bias legend: (A) Risk of bias due to confounding; (B) Risk of bias arising from measurement of the exposure; (C) Risk of bias in selection of participants into the study (or into the analysis); (D) Risk of bias due to post-exposure interventions; (E) Risk of bias due to missing data; (F) Risk of bias arising from measurement of the outcome; (G) Risk of bias in the selection of the reported result MD, mean difference References: [17,19,22,26]

BMI: A meta-analysis [17,19,22,26] was performed to evaluate differences in BMI between current cannabis users and nonusers, based on data from five observational studies. Cannabis users exhibited a significantly lower mean BMI, with a pooled MD of -1.85 kg/m² (95% CI: -2.38 to -1.32; p < 0.00001), as illustrated in Figure 5. High heterogeneity was observed across studies (I² = 100%), reflecting substantial methodological and population variability. Several studies also presented potential risks of selection and measurement bias. Consequently, while a significant association between cannabis use and lower BMI was identified, causality cannot be inferred.

Secondary Results

**Figure 6:**
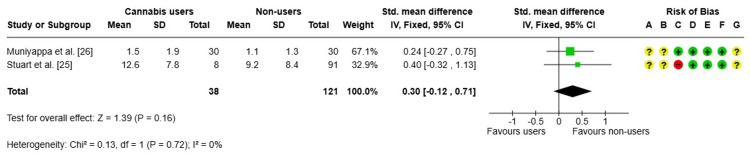
Forest plot and risk of bias of liver fat IV, inverse variance; Fixed, fixed-effects model; Std., standardized; + Low risk of bias; ? Some concerns; - High/very high risk of bias References: [25,26]

Liver fat (%): A meta-analysis [25,26] was conducted to evaluate the difference in liver fat levels between current cannabis users and nonusers, using data from two observational studies. The combined results revealed an SMD of 0.30, 95%CI (-0.12 to 0.71), which did not reach statistical significance (p = 0.16), as shown in Figure 6. Additionally, the included studies showed no heterogeneity (I² = 0%).

Although there is an apparent trend toward higher values in cannabis users, the current evidence does not allow us to conclude that there is a significant difference between the groups. The risk of bias analysis revealed minimal overall concern in most domains, though a high risk was identified in the selection of participants in one study and an uncertain risk in the measurement of exposure. The limited number of studies and small sample size limit the strength of the conclusions.

**Figure 7:**
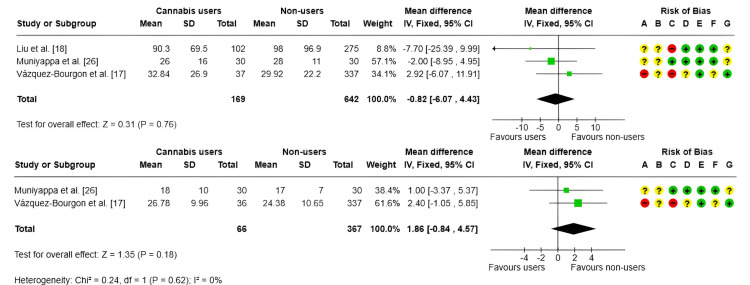
Forest plot and risk of bias: AST (1) and ALT (2) IV, inverse variance; Fixed, fixed-effects model; + Low risk of bias; ? Some concerns; - High/very high risk of bias ALT, alanine aminotransferase; AST, aspartate aminotransferase References: [17,18,26]

AST and ALT: For AST, the estimated MD was 1.86 units (95% CI: -0.84 to 4.57; p = 0.18) [17,26]. For ALT, the MD was -0.82 units (95% CI: -6.07 to 4.43; p = 0.76) [17,18,26], as shown in Figure 7. In both analyses, the CIs included the null value, indicating no statistically significant association. No heterogeneity was observed between studies (I² = 0%), supporting the consistency of these findings.

Overall, the results suggest that current cannabis use is not associated with significant changes in AST or ALT levels. However, some of the included studies presented risks of bias in specific domains, particularly regarding participant selection and exposure measurement.

**Figure 8:**
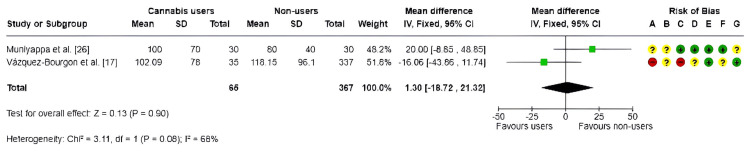
Forest plot and risk of bias of triglycerides IV, inverse variance; Fixed, fixed-effects model; + Low risk of bias; ? Some concerns; - High/very high risk of bias References: [17,25,26]

Triglycerides: A fixed-effects meta-analysis [17,26] was conducted to assess differences in triglyceride levels between current cannabis users and nonusers, based on two primary studies. The pooled MD was 1.30 mg/dL (95% CI: -18.72 to 21.32; p = 0.90), as shown in Figure 8, indicating no statistically significant differences between the groups.

Moderate heterogeneity was observed (I² = 68%), reflecting variability between the included studies. Furthermore, potential risks of bias were noted, particularly related to confounding factors and participant selection, which may have affected the internal validity of the results.

Sensitivity Analysis

A sensitivity analysis was conducted to evaluate the robustness of the findings and reduce the influence of studies with a high risk of bias. Excluding the studies by Vázquez-Bourgon et al. [17] and Adejumo et al. [24] yielded an OR of 0.66 (95% CI: 0.53-0.82; p = 0.0002), closely matching the initial estimate but indicating a slight attenuation of the effect on MASLD risk (Appendix A), thereby confirming the consistency of the results. A subgroup sensitivity analysis produced an overall OR of 0.64 (95% CI: 0.48-0.86; p = 0.0005), also consistent with the initial estimate, further supporting the robustness of the findings (Appendix B).

Publication Bias

**Figure 9:**
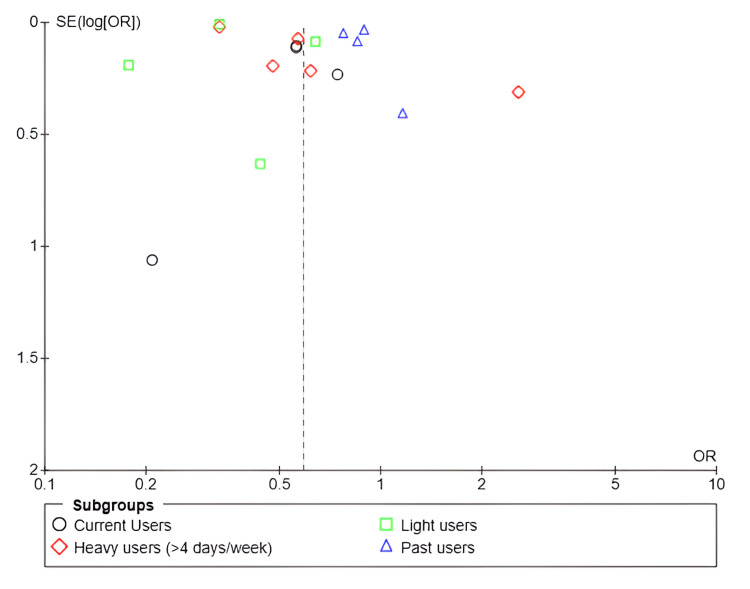
Funnel plot: subgroup MASLD risk MASLD, metabolic dysfunction-associated steatotic liver disease References: [17-24]

The funnel plot (Figure 9) demonstrated an asymmetric distribution of studies, suggesting the potential presence of publication bias or substantial heterogeneity in the results. Most studies, particularly those involving current, light, and past cannabis users, reported ORs below 1, supporting the hypothesis that cannabis use may confer a protective effect against hepatic steatosis. Nevertheless, the observed dispersion, especially among studies with lower precision, indicates that these findings should be interpreted with caution.

Risk of Bias

**Figure 10:**
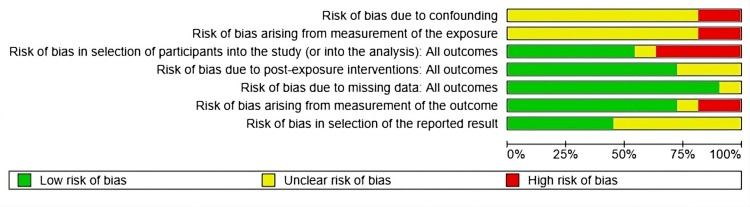
Risk of bias: each factor is presented as percentages overall among the included studies References: [17-26]

The risk of bias analysis (Figure 10) revealed significant methodological limitations in several domains, particularly regarding the control of confounding factors, the measurement of exposure, and participant selection. A substantial proportion of the included studies were classified as having a high or unclear risk of bias in these areas. In contrast, the domains related to subsequent interventions, missing data, and outcome measurement predominantly exhibited a low risk of bias; however, certain methodological uncertainties persisted.

Discussion

Cannabis use was associated with a lower risk of hepatic steatosis, as well as reduced FLI scores and BMI values. Light cannabis use demonstrated a significant protective effect against MASLD, whereas heavy use showed no significant association. Former users exhibited a modest reduction in risk. No statistically significant differences were observed in liver fat content, ALT, AST, or triglyceride levels, likely reflecting the limited number of studies included in these meta-analyses.

A study [27] revealed that, overall, cannabis use was not linked to liver stiffness. However, it was associated with a reduced risk of MASLD in women. A further meta-analysis [28] revealed that individuals who use cannabis have a lower BMI (25.5 vs. 27.5 kg/m²) and a reduced risk of obesity (AOR = 0.68). Individuals who are obese are particularly susceptible to MASLD, with a prevalence rate of 80% [29]. These results are similar to ours.

CBD may play a protective role in preventing hepatic steatosis through several molecular mechanisms. Suppression of NLRP3 inflammasome activation by deactivation of the NF-κB pathway in macrophages has been proposed as one of the most relevant anti-inflammatory mechanisms of CBD at the hepatic level [30].

Studies in vitro and in mouse models suggest that CBD and indole-3-carbinol can reduce fat accumulation in the liver by inhibiting lipogenesis and lipid storage, thereby helping to alleviate hepatic steatosis [31]. Furthermore, CBD has been found to reduce oxidative stress and selectively regulate intracellular signaling pathways that are involved in liver damage, such as JNK, without affecting p38 MAPK [32]. This suggests that it may have a hepatoprotective and metabolically beneficial effect.

Although public discourse often focuses on the potential therapeutic benefits of cannabis, the main scientific concerns are directed towards the physical, psychological, and social risks associated with its use, particularly when inhaled [33]. Cannabis smoke contains carcinogenic compounds at concentrations similar to or higher than those found in tobacco smoke, and cannabis use has been associated with respiratory conditions such as chronic bronchitis and epithelial damage [33]. Furthermore, the impact of the drug on the immune system has been observed, which is especially relevant for individuals with HIV/AIDS [33], as well as changes in cardiovascular [34] and reproductive functions [35].

Limitations

The review’s main limitation was the heterogeneity of methodology among the studies it included. Furthermore, the populations studied differed significantly. This variability introduced substantial differences in clinical and sociodemographic characteristics, which limited the generalizability of the findings.

Additional methodological limitations were identified, including the lack of standardization in the assessment of cannabis use, the absence of adequate control for relevant confounding variables, and the heterogeneity in diagnostic approaches for liver disease. Moreover, the potential influence of publication bias could not be excluded. These methodological constraints highlight the imperative need for well-designed longitudinal studies and rigorously conducted clinical trials to accurately evaluate the potential causal relationship between metabolic dysfunction and fatty liver disease.

## Conclusions

The results of this review indicate a potential relationship between cannabis use and the presence of fatty liver disease associated with metabolic dysfunction. However, such an association should be interpreted with caution for several reasons. First, there is methodological heterogeneity among the studies. Second, there is high variability in the populations analyzed. Third, there are inherent limitations of the predominant cross-sectional and retrospective observational designs.

While some studies report that cannabis may protect against hepatic steatosis, a direct causal relationship cannot be established. The lack of standardization in the diagnostic tools used, as well as differences in frequency, duration, and modality of use, limits the robustness of the conclusions.

## Figures and Tables

**Figure 11:**
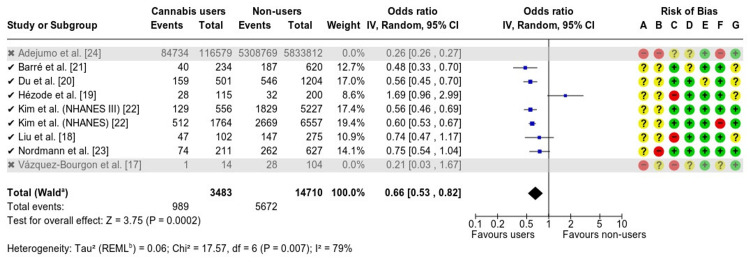
Forest plot and risk of bias: sensitivity analysis of MASLD risk IV, inverse variance; Random, random-effects model; + Low risk of bias; ? Some concerns; - High/very high risk of bias; ᵃ CI calculated using the Wald-type method; ᵇ Tau² calculated using the restricted maximum-likelihood method Risk of bias legend: (A) Risk of bias due to confounding; (B) Risk of bias arising from measurement of the exposure; (C) Risk of bias in selection of participants into the study (or into the analysis); (D) Risk of bias due to post-exposure interventions; (E) Risk of bias due to missing data; (F) Risk of bias arising from measurement of the outcome; (G) Risk of bias in selection of the reported result MASLD, metabolic dysfunction-associated steatotic liver disease References: [17-24]

**Figure 12:**
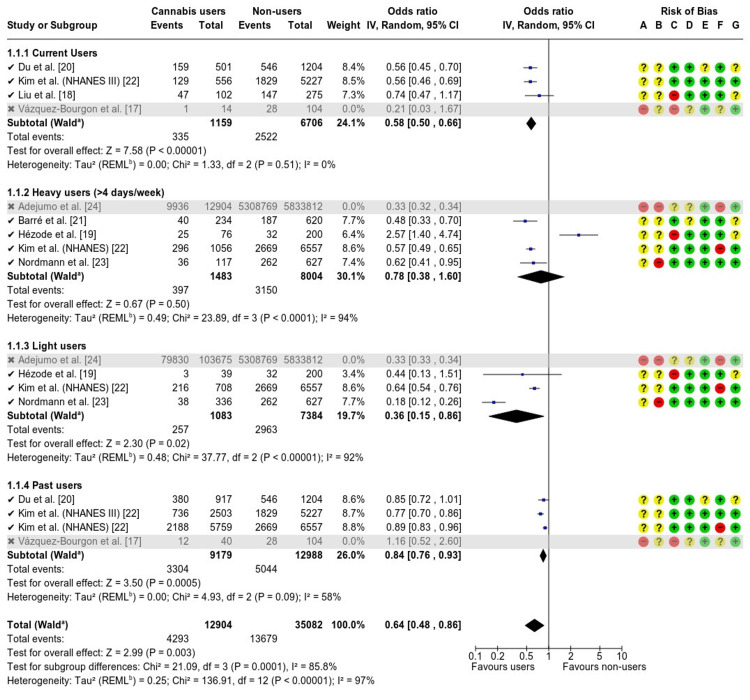
Forest plot and risk of bias: subgroup sensitivity analysis of MASLD risk IV, inverse variance; Random, random-effects model; + Low risk of bias; ? Some concerns; - High/very high risk of bias; ᵃ CI calculated using the Wald-type method; ᵇ Tau² calculated using the restricted maximum-likelihood method Risk of bias legend: (A) Risk of bias due to confounding; (B) Risk of bias arising from measurement of the exposure; (C) Risk of bias in selection of participants into the study (or into the analysis); (D) Risk of bias due to post-exposure interventions; (E) Risk of bias due to missing data; (F) Risk of bias arising from measurement of the outcome; (G) Risk of bias in selection of the reported result MASLD, metabolic dysfunction-associated steatotic liver disease References: [17-24]

## Appendices

Appendix A 

Appendix B
